# Repurposing piroxicam enhances the antineoplastic effects of docetaxel and enzalutamide in prostate cancer cells using 2D and 3D *in vitro* culture models

**DOI:** 10.3389/fcell.2025.1551010

**Published:** 2025-07-01

**Authors:** Amani Yehya, Fatima Ghamlouche, Raed Karami, Sana Hachem, Zahraa Salhab, Yen-Nien Liu, Georges Daoud, Wassim Abou-Kheir

**Affiliations:** ^1^ Faculty of Medicine, Department of Anatomy, Cell Biology and Physiological Sciences, American University of Beirut, Beirut, Lebanon; ^2^ International Ph.D. Program in Medicine, College of Medicine, Taipei Medical University, Taipei, Taiwan

**Keywords:** drug repurposing, prostate cancer, piroxicam, docetaxel, enzalutamide, tumor spheres, organoids

## Abstract

**Introduction:**

Drug repurposing is gaining consideration in cancer due to the challenges of poor outcomes and resistance associated with the current conventional modalities. Non-steroidal anti-inflammatory drugs (NSAIDs), widely used for treating inflammation, are being explored for their potential efficacy in cancer treatment, including prostate cancer (PCa). This study aims to evaluate the efficacy of Piroxicam (PXM), an NSAID, in enhancing the sensitivity of PCa cells to chemotherapy and hormonal drugs.

**Methods:**

Computational analysis was conducted to identify differentially expressed genes between our established murine PCa cell models, PLum-AD (androgen-dependent) and PLum-AI (androgen-independent), to uncover potential therapeutic targets. In two-dimensional (2D) cell culture, cell proliferation, viability, and migration assays were performed on PLum-AD and PLum-AI cells treated with PXM alone or in combination with docetaxel (Doc) or enzalutamide (Enz). Additionally, the impact of these treatments on stem-like progenitor cells was assessed using three-dimensional (3D)-Matrigel™-based sphere-forming and organoid formation assays.

**Results:**

Transcriptomic analysis revealed that inflammatory pathways are enriched during PCa progression, making them viable targets for NSAID-based interventions. Single treatment of PXM demonstrated significant anti-cancer effects on PLum-AD and PLum-AI cells, evidenced by reduced cell proliferation, viability, migration, sphere growth, and organoid growth.

**Discussion:**

Importantly, PXM treatment in combination with Doc or Enz resulted in more pronounced antineoplastic effects compared to single-drug exposure. Our work suggests PXM as a potential adjunctive therapy to enhance the efficacy of conventional treatments in PCa patients.

## 1 Introduction

Prostate cancer (PCa) denotes a major health concern where it ranks as the second most frequently diagnosed malignancy and the fifth leading cause of cancer-related mortalities among men worldwide (Sung et al., 2021). PCa is usually indolent and develops slowly as androgen-dependent tumor that can be easily managed (Aurilio et al., 2020). However, most PCa tumors escape androgen manipulation and progress into an aggressive hormone-refractory stage known as metastatic castration-resistant PCa (mCRPC), where the tumor becomes insensitive to androgen. At this stage, PCa is characterized with significant morbidity and poor prognosis (Ehsani et al., 2021; Le et al., 2023). Treatment modalities targeting PCa vary based on the staging and clinical advancement of the disease and include surgical intervention, radiotherapy, and systemic therapies (Swami et al., 2020). Despite the efforts that have been made to mitigate the symptoms of this disease and enhance the survival expectancy of PCa patients, there are currently no curable options available. Thus, novel therapeutics are needed to improve clinical outcomes of mCRPC.

PCa is driven by pre-cancerous lesions designated as prostatic intraepithelial neoplasia (PIN) that surround inflammatory cells. PCa then progresses from PIN into locally invasive androgen-dependent adenocarcinoma and ultimately advances into mCRPC (Kessel et al., 2021). Compelling evidence suggests that chronic inflammation is a prominent risk factor associated with the development of PIN, eventually leading to PCa (de Bono et al., 2020; Fernandes et al., 2023). In fact, chronic prostatic inflammation was correlated with increased high grade PCa (Göbel et al., 2021). Similarly to other cancer types, inflammatory markers are often detected in the tumor microenvironment (TME) of PCa (Bahmad et al., 2021a). Indeed, studies indicate that cytokines are involved in several aspects of PCa pathogenesis including cancer cells proliferation, angiogenesis, metastasis, and epithelial-mesenchymal transition (EMT) among others (Mughees et al., 2022).

Drug repurposing, also termed as drug repositioning, has gained a lot of interest in recent years as a treatment approach against cancer including PCa. This novel approach refers to the use of preexisting clinically approved drugs for applications different than the ones they were originally designed for (Xia et al., 2024a). Using well-established drugs with known side effects accelerates the drug development process by providing a safer and cheaper approach to study anti-cancer compounds as compared to *de novo* synthesis of new drugs with unknown pharmacokinetics and toxicity (Bahmad et al., 2021b).

Non-steroidal anti-inflammatory drugs (NSAIDs) are a family of compounds ubiquitously prescribed for their analgesic, anti-pyretic, anti-thrombotic, and anti-inflammatory properties (Kaduševičius, 2021). NSAIDs work by reversibly targeting cyclooxygenase (COX) enzymes and inhibiting the production of prostaglandins (PGs), which are key players involved in inflammatory processes (Panchal and Prince, 2023). Several studies indicate that NSAIDs exhibit preventive and anti-cancer effects against various cancer types including colorectal, breast, head and neck, and prostate cancers (Zappavigna et al., 2020; Kolawole and Kashfi, 2022; Burn et al., 2020; Balamurugan et al., 2023; Hedberg et al., 2019).

Piroxicam (PXM) is a non-selective COX inhibitor that belongs to the enolic acid derivatives of NSAIDs (Xu et al., 2014). The anti-inflammatory role of NSAIDs, including PXM, has been investigated in tumorigenesis across multiple cancers; however, the vast majority of publications refer to preclinical studies. Notably, PXM was extensively studied in colorectal cancer, where it exhibited anti-angiogenic role and reduced tumor size in animal models (Saini and Sanyal, 2014). Its anti-cancer effect was also reported in *in vitro* breast cancer models through reactive oxygen species-mediated apoptosis (Rai et al., 2015), as well as in bladder cancer cell lines, where it inhibited proliferation as a single agent and in combination with carboplatin (Silva et al., 2017a).

Clinically, a Phase IIb clinical trial in patients with a history of resected colorectal adenomas demonstrated that PXM significantly reduced rectal mucosa PGE2 levels, a biomarker of colorectal tumor progression (Calaluce et al., 2000). However, the study reported gastrointestinal toxicity, which limited its long-term application in cancer prevention (Calaluce et al., 2000). Another NSAID, celecoxib, a selective COX-2 inhibitor, has been evaluated in several clinical trials for its potential anti-cancer effects. In familial adenomatous polyposis (FAP), Thompson *et al.* demonstrated that administering 400 mg/day celecoxib for 5–23 months reduced adenoma recurrence in high-risk FAP patients (Thompson et al., 2016). In gastric cancer, Guo *et al.* conducted a randomized, case–control, multi-center clinical trial in which patients with advanced gastric cancer received daily celecoxib combined with chemotherapy for 5 months. The results showed that celecoxib, as an adjuvant to chemotherapy, enhanced overall survival, disease-free survival, progression-free survival, and quality of life in COX-2 positive gastric cancer patients (Guo et al., 2017). In a subsequent study, the same research group investigated celecoxib in patients with metastatic or postoperative recurrent advanced gastric cancer, further supporting its potential clinical application (Guo et al., 2019).

Given its promising potency in several cancer types, we aimed in our study to investigate the efficacy of PXM, alone and in combination with chemotherapeutic and hormonal drugs, on murine PCa cells *in vitro.* Our results demonstrated that PXM as a single agent or when combined with docetaxel (Doc) and enzalutamide (Enz) reduces the proliferation, viability, and migration of PCa cells *in vitro*. Importantly, our study revealed for the first time that PXM, alone and in combination with PCa conventional therapeutics, effectively targets the stem/progenitor PCa cells using *in vitro* three-dimensional (3D) cell cultures. To our knowledge, this is the first study to demonstrate PXM’s potential to enhance the efficacy of traditional anti-cancer treatments. Overall, our research positions PXM as a promising candidate for repurposing as an anti-cancer agent and provides compelling evidence supporting its consideration as an adjunctive therapy for PCa.

## 2 Materials and methods

### 2.1 Cell culture

PLum-AD and PLum-AI cell lines are novel murine *in vitro* models earlier generated in our laboratory from androgen-dependent (PLum-AD cells) and androgen-independent (PLum-AI cells) PCa (Daoud et al., 2016; Abou-Kheir et al., 2011). PLum-AD cells were derived from prostate adenocarcinoma tumors and exhibit an epithelial phenotype, whereas PLum-AI cells were derived from tumors undergoing EMT. Moreover, both cell types have the same defined genetic background of *Pten* ^*-/−*^ *TP53* ^*−/−*^. Thus, these cells are useful models that recapitulate the progression of PCa from the primary androgen-dependent state to the more invasive androgen-independent phenotype upon androgen deprivation.

PLum-AD and PLum-AI cells were cultured in advanced Dulbecco’s Modified Eagle Medium/Ham’s F-12 (adDMEM/F12) medium (Gibco^TM^, ThermoFisher Scientific) containing 1% Penicillin/Streptomycin (Sigma-Aldrich), 0.2% Plasmocin^TM^ prophylactic (Invivogen), 1% Hepes (Gibco), 1% Glutamax (Gibco), and 10 ng/mL epidermal growth factor (EGF) (R&D Systems). Cells were incubated in a humidified atmosphere and 5% CO_2_ at 37°C and maintained *mycoplasma* free.

### 2.2 Drugs preparation

PXM (Sigma-Aldrich), Doc (BOC Sciences), and Enz (Biorbyt) were reconstituted in dimethylsulfoxide (DMSO) at stock concentrations of 150 mM, 10 mM, and 40 mM, respectively. Drugs were stored at −20°C.

### 2.3 Thiazolyl blue tetrazolium bromide cell proliferation assay

To determine the sole and combinatory effects of PXM, Doc, and Enz on the proliferation of PLum-AD and PLum-AI cells, 3-(4, 5-dimethylthiazol-2-yl)-2, 5-diphenyltetrazolium bromide (MTT) (Sigma-Aldrich) assay was used. Cells were seeded in triplicates in 96-well culture plates and incubated overnight. Then, cells were treated with increasing concentrations of PXM (250, 500, and 750 μM), Doc (1, 5, and 10 nM), and Enz (25, 50, 100 μM) for 24, 48, and 72 h. At each time point, media was aspirated from all the wells and fresh media supplemented with 5 mg/mL MTT reagent (in PBS) was added. Following 3 h incubation at 37°C in 5% CO_2_, media was discarded and replaced with absolute isopropanol (Sigma-Aldrich). Optical density (OD) was measured using the Tristar Multimode Reader (BERTHOLD) at 595 nm. Cell proliferation was determined and represented as the percentage of OD ratio of the treated groups over the control.

### 2.4 Trypan blue exclusion assay

To assess the effects of the single and combination treatments of PXM, Doc, and Enz on the viability of PLum-AD and PLum-AI cells, trypan blue exclusion assay was employed. Briefly, cells were seeded in duplicates in 24-well culture plates. On the following day, cells were treated with various concentrations of PXM (250 and 500 μM), Doc (1 and 5 nM), and Enz (25 and 50 nM) for 24 and 48 h. At each time point, attached cells were harvested and a mix of cell suspension and trypan blue dye at a 1:1 ratio was prepared. Viable cells were counted using a hemocytometer under an inverted light microscope. Cell viability was determined as the percentage of the number of viable cells ratio of the treated groups over the control.

### 2.5 Wound healing assay

To determine the effects of PXM, Doc, and Enz, alone or in combination, on the directional cell migration ability of PLum-AD and PLum-AI cells, wound healing or scratch assay was used. Accordingly, a confluent monolayer of cells, that were seeded in 24-well culture plates, were treated with 10 μg/mL mitomycin C (Sigma-Aldrich) for 20 min to block the cellular proliferation. Then, a uniform scratch in each well was made using sterile 200 μL micropipette tips. Cells were washed with 1X Dulbecco’s phosphate buffered saline (D-PBS) (Sigma-Aldrich) to remove the detached cells and cellular debris, after which the remaining cells were treated with PXM (250 and 500 μM), Doc (1 and 5 nM), and Enz (25 and 50 nM). Images of the scratches were subsequently acquired using Axiovert microscope from Zeiss (San Diego, CA, USA) at 0, 4, 8, 12, and 16 h and 0, 4, 8, 12, 16, and 24 h post-treatments for PLum-AD and PLum-AI cells, respectively. Wound areas for each condition were evaluated using ImageJ tool and the percentage of wound area at the different time points was calculated by dividing the area % at time zero by the area % at the specific time point and multiplying by 100.

### 2.6 Sphere formation assay

To assess the effectiveness of PXM in targeting PCa stem/progenitor, whether alone or in combination with the used PCa conventional therapeutic agents, *in vitro* three-dimensional (3D) sphere formation assay was employed and the effects of the treatments on prostatosphere forming-ability of PLum-AD and PLum-AI cells were determined. The sphere formation assay was performed as previously described by our group (Bahmad et al., 2018). In brief, single PLum-AD and PLum-AI cells were suspended in cold Matrigel^TM^ and serum-free medium at 1:1 ratio. 10 μL of the cell/Matrigel^TM^ suspension was seeded around the rim of each well of a 96-well cell culture plate and kept solidifying for 30 min at 37°C in a humidified incubator. Then, culture medium with or without corresponding treatments was added gently in the middle of each well. Media was replenished every other day as per the day of seeding. After 7–8 and 4–5 days of PLum-AD and PLum-AI seeding, respectively, the formed spheres were counted, and bright-field images of the spheres were captured using Axiovert microscope from Zeiss (San Diego, CA, USA). The percentage of sphere formation unit (SFU) was evaluated by dividing the number of counted spheres per condition by the original number of seeded cells and multiplying by 100. The average diameter of at least 30 spheres/condition was determined using Carl Zeiss Zen 2012 image software.

### 2.7 Cell-line derived organoid formation assay

The cell-line derived organoids formation assay was performed with modifications from the previously described methodology for the patient-derived organoids (Cheaito et al., 2020). Briefly, single PLum-AD and PLum-AI cells were suspended in cold Matrigel^TM^ and serum-free medium at 9:1 ratio. A 5 μL of the cell/Matrigel^TM^ suspension was seeded as a drop in the middle of each well in 96-well culture plate and kept solidifying for 30 min at 37°C in a humidified incubator. Subsequently, culture medium containing the growth factors listed in Table 1, with or without the corresponding treatments, was added gently to each well. The medium was replenished every other day from the day of seeding. After 7 to 8 and 4–5 days of PLum-AD and PLum-AI seeding, respectively, the formed organoids were counted, and bright-field images of the organoids were captured using Axiovert microscope from Zeiss at ×10 magnification. The OFC was calculated as the number of formed organoids starting with the same number of seeded cells for the different conditions.

**TABLE 1:** Overview of culture medium components for mouse prostate organoids.

Growth Factor	Stock concentration	Final concentration
B27	50x	1x
NAC	500 mM (in PBS)	1.25 mM
EGF	500 μg/mL	50 ng/mL
NOG	100 μg/mL (in PBS + 0.1% BSA)	100 ng/mL
R-spondin	500 μg/mL (in PBS + 0.1% BSA)	500 ng/mL
A83-01	5 mM in DMSO	200 nM
DHT	1 µM in ethanol	1 nM

NAC, N-acetylcysteine; EGF, epidermal growth factor; NOG, noggin; DHT, dihydrotestosterone.

### 2.8 Transcriptomic data analysis and protein-protein network

Microarray data for murine PCa cell lines derived from androgen-dependent (PLum-AD cells) and androgen-independent (PLum-AI cells) PCa were previously deposited in NCBI’s Gene Expression Omnibus (GEO) under the GEO Series accession number GSE151187 (Daouk et al., 2020). Data was analyzed using GEO2R–GEO tool to derive the differentially expressed genes (DEGs) between PLum-AD and PLum-AI groups with significance level cut-off and Log 2-fold change threshold set at 0.01 and 2, respectively. DEGs enrichment analysis, which included the top 500 most relevant genes, was executed using the Enrichr tool MSigDB Hallmark 2020 database. Subsequently, genes enriched in pathways and cellular processes that are relevant to this study and related to PCa development and progression were elected. As such, a list of genes enriched in EMT, tumor necrosis factor-alpha (TNFα) signaling via NF-kB, interferon-gamma response, inflammatory response, and IL-6/JAK/STAT3 signaling was determined. Functional protein association network to map the protein-protein interactions between the selected DEG products in *mus musculus* organisms was established using STRING tool (version 12.0) (Szklarczyk et al., 2023).

### 2.9 Statistical analysis

Statistical analysis was performed using GraphPad Prism 8 (version 8.0.2; GraphPad Software Inc., La Jolla, CA, United States). Data was expressed as mean ± standard error of the mean (SEM) of at least three independent experiments. Results were analyzed using One-way or Two-way ANOVA tests comparing the mean between the distinct experimental groups. Differences were considered significant if *P* < 0.05.

## 3 Results

### 3.1 Transcriptomic data and functional protein association network analysis implicate inflammatory pathways in the progression of PCa

**FIGURE 1:**
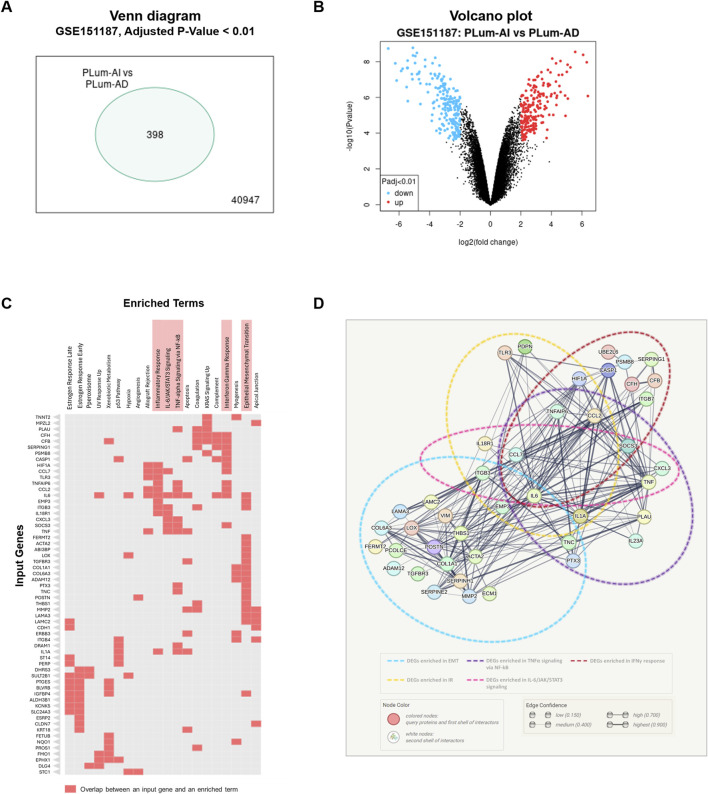
Inflammatory and epithelial-mesenchymal transition pathways are implicated in murine PCa progression. **(A)** Venn diagram representing the significantly differentially expressed genes (DEGs) between murine PLum-AI and PLum-AD PCa cells obtained through analysis of GSE151187 microarray data using GEO2R–GEO tool (Adjusted p-value and Log 2-fold change threshold set at 0.01 and 2, respectively; **(B)** Volcano plot representing the downregulated (blue dots) and upregulated (red dots) genes between PLum-AI and PLum-AD cells obtained through analysis of GSE151187 microarray data using GEO2R–GEO tool (Adjusted p-value and Log 2-fold change threshold set at 0.01 and 2, respectively; **(C)** Clustergram representing the enriched terms obtained by pathway analysis of the DEGs in the murine PCa cells using the Enrichr tool MSigDB Hallmark 2020 database; **(D)** Protein-protein interaction network for DEGs derived from the selected pathways of relevance to PCa and inflammation that included epithelial-mesenchymal transition, interferon gamma response, TNF-alpha signaling via NF-kB, IL-6/JAK/STAT3 signaling, and inflammatory response. Functional protein association network was established using STRING tool (version 12.0). DEGs: differentially expressed genes; EMT: epithelial-mesenchymal transition; TNFα: tumor necrosis factor-alpha; NF-kB: nuclear factor kappa-light-chain-enhancer of activated B cells; IFNγ: interferon-gamma; IR: inflammatory response.

To decipher the mechanisms underlying the progression of PCa from the primary androgen-dependent state to the aggressive androgen-independent, transcriptomic analysis was performed on previously obtained microarray hybridization data deposited in GEO under the accession number GSE151187 (Daouk et al., 2020). Data analysis using GEO2R–GEO tool revealed 398 DEGs between PLum-AI and PLum-AD cells (Figures 1A,B; Supplementary Tables 1, 2). The significantly upregulated and downregulated genes identified in PLum-AI compared to PLum-AD cells were interrogated using the Enrichr tool MSigDB Hallmark 2020 database to identify enriched pathways and cellular processes related to these DEGs. Interestingly, data analysis showed that the DEGs between the murine PCa cells were associated with several pathways related to inflammation as well as EMT (Figure 1C; Supplementary Table 3). Besides, several deregulated genes between PLum-AI and PLum-AD cells, presented in the Clustergram generated by Enrichr tool, such as *PTGES*, *IL6*, *HIF1A*, *CCL7*, *TLR3*, *CCL2*, *TNF*, and *IL1A* (all *p* < 0.02) are related to prostaglandins synthesis and their downstream signaling (Shen and Li, 2017; Pahl et al., 2007; Liu et al., 2018; Salvi et al., 2016; Finetti et al., 2020; Hinson et al., 1996; Dossus et al., 2010; Ja et al., 2021; Wang et al., 2022) (Figure 1C). Moreover, the protein-protein network interaction analysis involving the DEGs suggested a possible interaction between signaling molecules related to diverse inflammatory pathways with IL6 being a common player to all (Figure 1D).

The transcriptomic and protein-protein network analysis reveals an enrichment in the pathways related to inflammation during the progression of PCa. Thus, these results indicate that PCa may be vulnerable to NSAIDs that inhibit the inflammatory cascades and may consequently hinder PCa development and progression.

### 3.2 PXM reduced the proliferation of murine PCa cells alone and in combination with Doc or Enz

**FIGURE 2:**
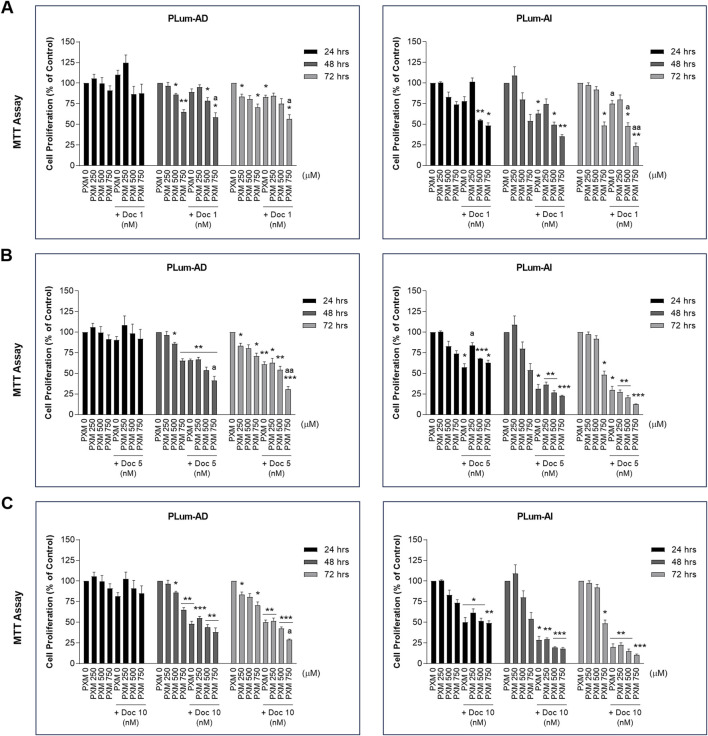
Increasing concentrations of piroxicam, alone or in combination with docetaxel, reduced the proliferation of murine prostate cancer cells. The antiproliferative effect of 250, 500, and 750 μM of piroxicam on PLum-AD and PLum-AI cells alone or in combination with 1 **(A)**, 5 **(B)**, and 10 **(C)** nM of docetaxel was assessed in triplicates using thiazolyl blue tetrazolium bromide (MTT) assay at 24, 48, and 72 h post-treatment. Results are expressed as a percentage of the treated groups compared to the control at each time point and data are reported as mean ± SEM of at least three independent experiments (*P < 0.05; **P < 0.01; ***P < 0.001 indicate significance when comparing to the untreated group and a *P* < 0.05; aa *P* < 0.01 indicate significance when comparing the piroxicam/docetaxel combination treated groups to docetaxel treated group). PXM: piroxicam; Doc: docetaxel.

**FIGURE 3:**
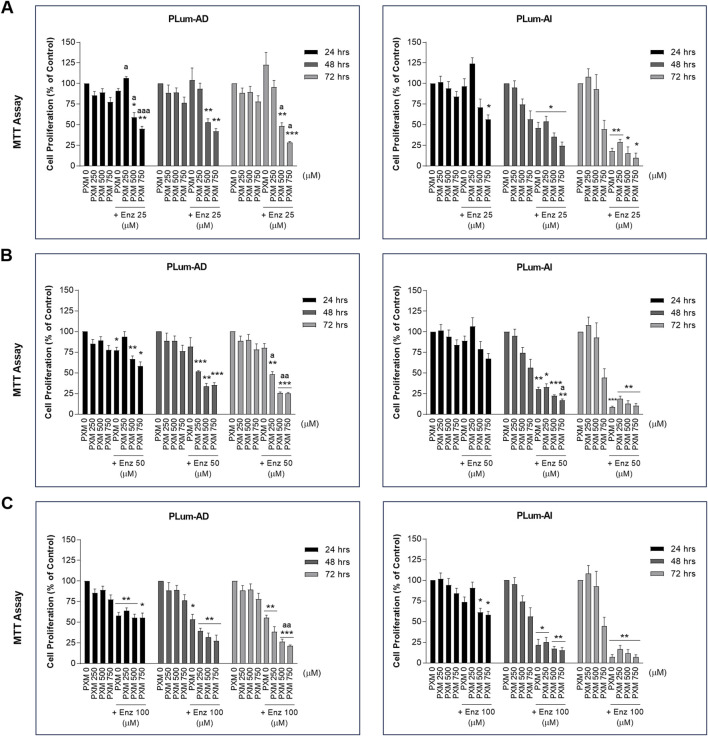
Increasing concentrations of piroxicam, alone or in combination with enzalutamide, reduced the proliferation of murine prostate cancer cells. The antiproliferative effect of 250, 500, and 750 μM of piroxicam on PLum-AD and PLum-AI cells alone or in combination with 25 **(A)**, 50 **(B)**, and 100 **(C)** μM of enzalutamide was assessed in triplicates using thiazolyl blue tetrazolium bromide (MTT) assay at 24, 48, and 72 h post-treatment. Results are expressed as a percentage of the treated groups compared to the control at each time point and data are reported as mean ± SEM of at least three independent experiments (*P < 0.05; **P < 0.01; ***P < 0.001 indicate significance when comparing to the untreated group and a *P* < 0.05; aa *P* < 0.01; aaa *P* < 0.001 indicate significance when comparing the piroxicam/enzalutamide combination treated groups to enzalutamide treated group). PXM: piroxicam; Enz: enzalutamide.

To verify the effect of PXM, alone or in combination with PCa conventional therapeutics, on the cell growth of PLum-AD and PLum-AI cells, MTT assay was used. Accordingly, murine PCa cells were treated with increasing concentrations of PXM (250, 500, and 750 μM) as a single agent or added to various concentrations of either Doc or Enz for up to 72 h. PXM alone significantly reduced the proliferation of both cell lines, PLum-AD and PLum-AI, in a dose-dependent manner (Figures 2, 3). The decrease in the growth of PLum-AD cells reached approximately 25% upon treatment with 750 μM of PXM for 72 h. Interestingly, a more prominent decrease in cell proliferation was observed in PLum-AI cells, reaching 53%, when treated with the same concentration of PXM and at the same time point (Figures 2, 3).

Each of the tested concentrations of PXM (250, 500, and 750 μM) were added to different concentrations of either Doc or Enz ranging from 1 to 10 nM and 25 to 100 μM, respectively. PXM significantly enhanced the effects of Doc and Enz on the murine PCa cells (Figures 2, 3). Specifically, 750 μM of PXM significantly increased the effect of 1 nM of Doc starting 48 h post-treatment. This combinatorial treatment decreased the cellular proliferation of PLum-AD and PLum-AI cells by almost two folds compared to the effect of 1 nM of Doc alone (Figure 2A). On the other hand, treatment with PXM starting 500 μM significantly enhanced the cytotoxicity of all the tested Enz concentrations on PLum-AD cells at 72 h (Figures 3A–C). While 750 μM of PXM significantly enhanced the growth inhibitory effect of 50 μM of Enz at 48 h on PLum-AI cells (Figure 3B).

These results indicate that PXM alone reduced the proliferation of murine PCa cells and enhanced the cytotoxic effects of PCa conventional therapeutic agents.

### 3.3 PXM alone decreased the viability of murine PCa cells and synergized with Doc and Enz in reducing murine PCa cell viability

**FIGURE 4:**
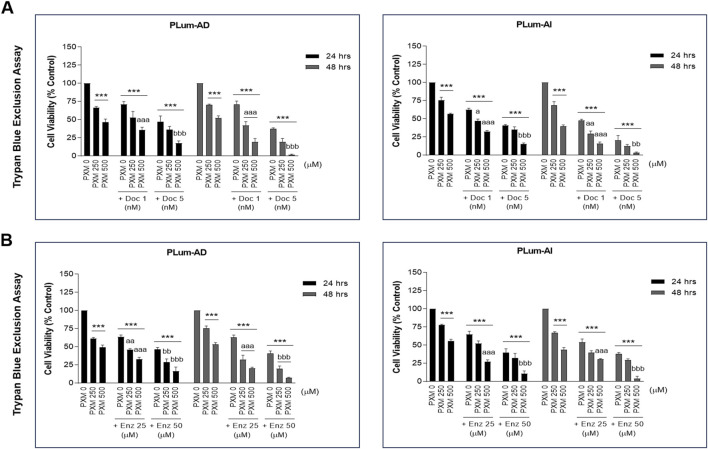
Increasing concentrations of piroxicam, alone or in combination with docetaxel or enzalutamide, reduced the viability of murine prostate cancer cells in a dose-dependent manner. The effect of 250 and 500 μM of PXM on the viability of PLum-AD and PLum-AI cells alone or in combination with 1 and 5 nM of docetaxel **(A)** or 25 and 50 μM of enzalutamide **(B)** was determined in duplicates using the trypan blue exclusion assay at 24 and 48 h post-treatment. Results are expressed as a percentage of the treated groups compared to the control at each time point and data are reported as mean ± SEM of three independent experiments (***P < 0.001 indicates significance when comparing to the untreated group, a *P* < 0.05; aa *P* < 0.01; aaa *P* < 0.001 indicate significance when comparing the piroxicam/1 nM of docetaxel and piroxicam/25 μM of enzalutamide combination treated groups to 1 nM of docetaxel and 25 μM of enzalutamide treated group, respectively, **P < 0.01; ***P < 0.001 indicate significance when comparing the piroxicam/5 nM of docetaxel and piroxicam/50 μM of enzalutamide combination treated groups to 5 nM of docetaxel and 50 μM of enzalutamide treated group, respectively). PXM: piroxicam; Doc: docetaxel; Enz: enzalutamide.

To confirm the MTT results and to assess the effect of PXM on cell viability, alone or in combination with PCa standard therapeutic agents, the trypan blue exclusion dye assay was employed. Indeed, with comparable trends to the MTT assay, treatment with 250 and 500 μM of PXM significantly reduced the cell viability of both murine cell lines 24 and 48 h post-treatment in a dose-dependent manner (Figures 4A,B). PXM treatment alone, up to 500 μM, reduced the cell viability of PLum-AD and PLum-AI cells by approximately 50% compared to the untreated cells at both time points (Figures 4A,B).

Interestingly, when these two concentrations of PXM were combined with either Doc or Enz at all assessed concentrations (1 or 5 nM of Doc and 25 or 50 μM of Enz), the viability of PLum-AD and PLum-AI cells was further significantly decreased when compared to the effects of each single drug (Figures 4A,B). In fact, combining 500 μM of PXM with either 5 nM of Doc or 50 μM of Enz led to almost a total eradication of the murine PCa cells (Figures 4A,B). To confirm a possible synergistic effect when combining PXM with PCa conventional therapeutic agents, we analyzed the 48 h treatment results obtained by the trypan blue exclusion assay using the computational Compusyn tool. Remarkably, synergy was observed in all the conducted combinations with a combination index (CI) less than 1 (Tables 2, 3). Very strong synergism (CI < 0.1) was detected when 500 μM of PXM was combined with 5 nM of Doc in PLum-AI cells (Table 2) and strong synergism (CI between 0.1 and 0.3) was observed when 500 μM of PXM was combined with 50 μM of Enz in PLum-AI cells (Table 3) (Chou, 2008).

**TABLE 2:** Piroxicam and docetaxel Compusyn study on murine prostate cancer cells.

Cell Line	PXM (μM)	Doc (nM)	CI
PLum-AD cells	250	1	0.59695
	250	5	0.48360
	500	1	0.33084
	500	5	0.04235
PLum-AI cells	250	1	0.76658
	250	5	0.69698
	500	1	0.63595
	500	5	2.24E-6

Compusyn analysis was computed on piroxicam and docetaxel-treated murine PCa, treated cells at 48 h post-treatment. Combination index values of CI, 1 additive effect, CI < 1 synergistic, and CI > 1 antagonistic. Red values indicate very strong synergism. PXM, piroxicam; Doc, docetaxel; CI, combination index.

**TABLE 3:** Piroxicam and enzalutamide Compusyn study on murine prostate cancer cells.

Cell Line	PXM (μM)	Enz (μM)	CI
PLum-AD cells	250	25	0.65446
	250	50	0.61838
	500	25	0.58480
	500	50	0.33535
PLum-AI cells	250	25	0.97326
	250	50	0.96854
	500	25	0.99234
	500	50	0.16305

Compusyn analysis was computed on piroxicam and enzalutamide-treated murine PCa, treated cells at 48 h post-treatment. Combination index values of CI, 1 additive effect, CI < 1 synergistic, and CI > 1 antagonistic. Red values indicate strong synergism. PXM, piroxicam; Enz, enzalutamide; CI, combination index.

### 3.4 PXM reduced the migration ability of murine PCa cells alone and in combination with Doc or Enz

**FIGURE 5:**
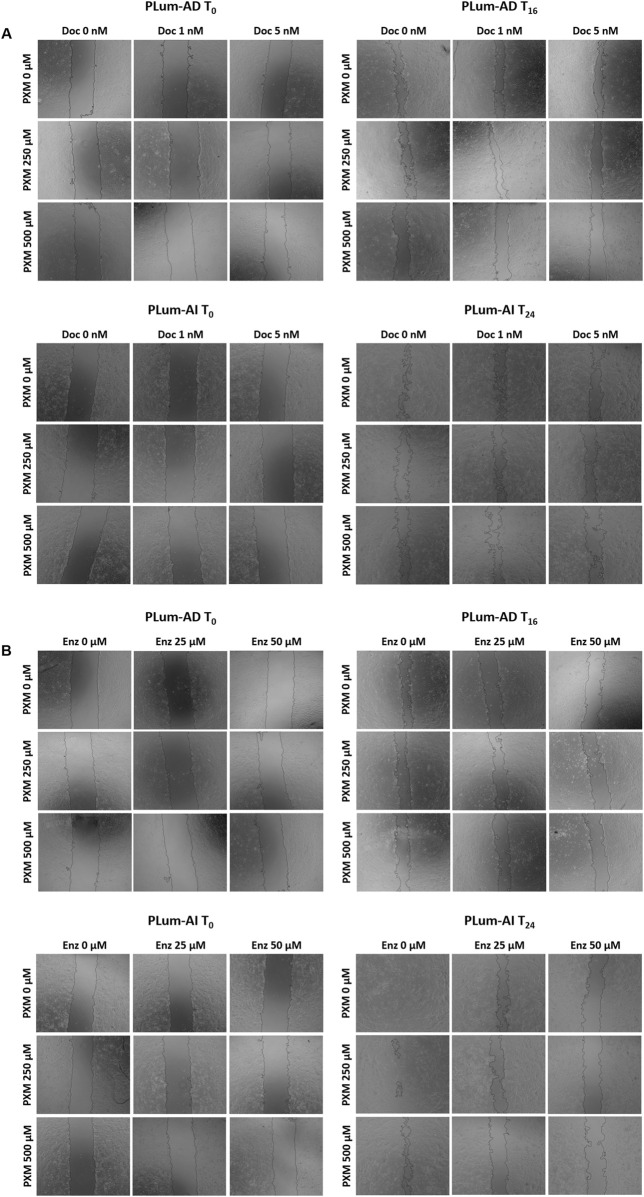
Piroxicam, alone or in combination with docetaxel or enzalutamide, reduced the migration indices of murine prostate cancer cells. Representative bright-field images of the wounds in PLum-AD at 0 h and 16 h and in PLum-AI cells at 0 h and 24 h post-treatment with PXM alone or in combination with docetaxel **(A)** or enzalutamide **(B)**. Images were acquired using Axiovert microscope from Zeiss at ×5 magnification. PXM: piroxicam; Doc: docetaxel; Enz: enzalutamide; T: hours post-treatment.

**FIGURE 6:**
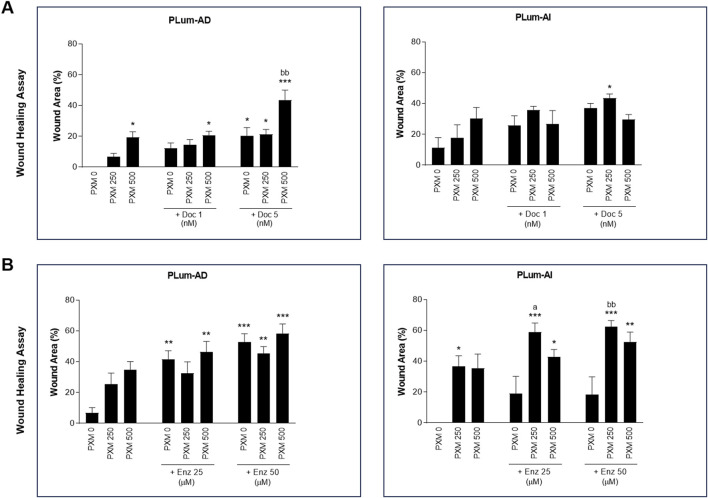
Increasing concentrations of Piroxicam, alone or in combination with docetaxel or enzalutamide, reduced the migration indices of murine prostate cancer cells. The effect of 250 and 500 μM of PXM on the migration of PLum-AD and PLum-AI cells alone or in combination with 1 and 5 nM of docetaxel **(A)** or 25 and 50 μM of enzalutamide **(B)** was determined using the wound healing assay. A uniform scratch was made in PLum-AD and PLum-AI confluent monolayer of cells using 200 μL micropipette tips. The wound area was monitored over time and serial images were captured at ×5 magnification at 0 h and up to 16 and 24 h post-treatment for PLum-AD and PLum-AI, respectively. Quantification of the open wound area was assessed over time using ImageJ tool. For each group, results are presented as the percentage of wound area at 16 h for PLum-AD cells and 24 h for PLum-AI cells over the percentage of wound area at time zero. Data are reported as mean ± SEM of four independent experiments (*P < 0.05; **P < 0.01; ***P < 0.001 indicate significance when comparing to the untreated group, a *P* < 0.05 indicates significance when comparing the piroxicam/1 nM of docetaxel and piroxicam/25 μM of enzalutamide combination treated groups to 1 nM of docetaxel and 25 μM of enzalutamide treated group, respectively, bb *P* < 0.01 indicates significance when comparing the piroxicam/5 nM of docetaxel and piroxicam/50 μM of enzalutamide combination treated groups to 5 nM of docetaxel and 50 μM of enzalutamide treated group, respectively). PXM: piroxicam; Doc: docetaxel; Enz: enzalutamide.

To assess the effect of PXM, alone or in combination with either Doc or Enz, on the migration of murine PCa cells, wound healing assay was employed. Treatment with PXM alone significantly delayed the migration of PLum-AD and PLum-AI cells compared to the untreated cells (Figures 5, 6). The percentage of wound area in 250 and 500 μM-PXM treated PLum-AD and PLum-AI groups was approximately 20% and 40%, respectively, whereas the untreated groups exhibited a complete wound closure 16 and 24 h post-treatment (Figure 6).

Interestingly, when PXM was combined with 5 nM of Doc or 25 and 50 μM of Enz, the migration ability of PLum-AD and PLum-AI cells was significantly reduced with enhanced effects compared to either Doc or Enz alone (Figures 5, 6). In fact, 250 μM of PXM combined with Enz significantly hindered the migration of the invasive PLum-AI cells when compared to the effect of Enz alone, whereby the percentage of wound area was 80% in the combination group compared to almost 20% in the Enz treated cells 24 h post-treatment (Figure 6B).

### 3.5 PXM reduced the growth of murine prostatospheres alone and in combination with Doc or Enz

**FIGURE 7:**
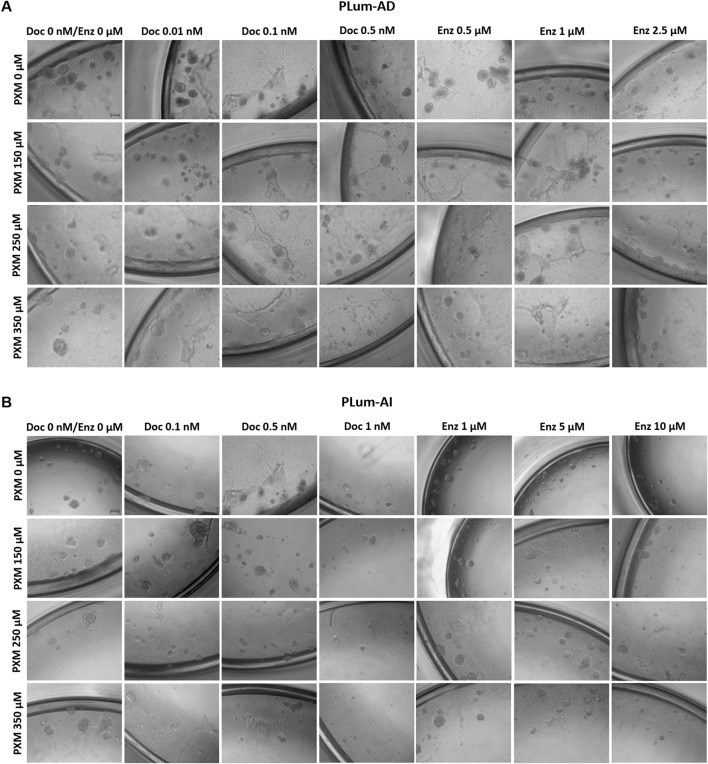
Piroxicam, alone or in combination with docetaxel or enzalutamide, reduced the growth of murine prostatospheres. Representative images of PLum-AD **(A)** and PLum-AI **(B)** cells-derived spheres treated with piroxicam and docetaxel or enzalutamide after 7 to 8 and 4–5 days post-seeding, respectively, are shown (scale bar = 100 μm). Images were acquired using Axiovert inverted microscope equipped with Carl Zeiss Zen 2012 image software. PXM: piroxicam; Doc: docetaxel; Enz: enzalutamide.

**FIGURE 8:**
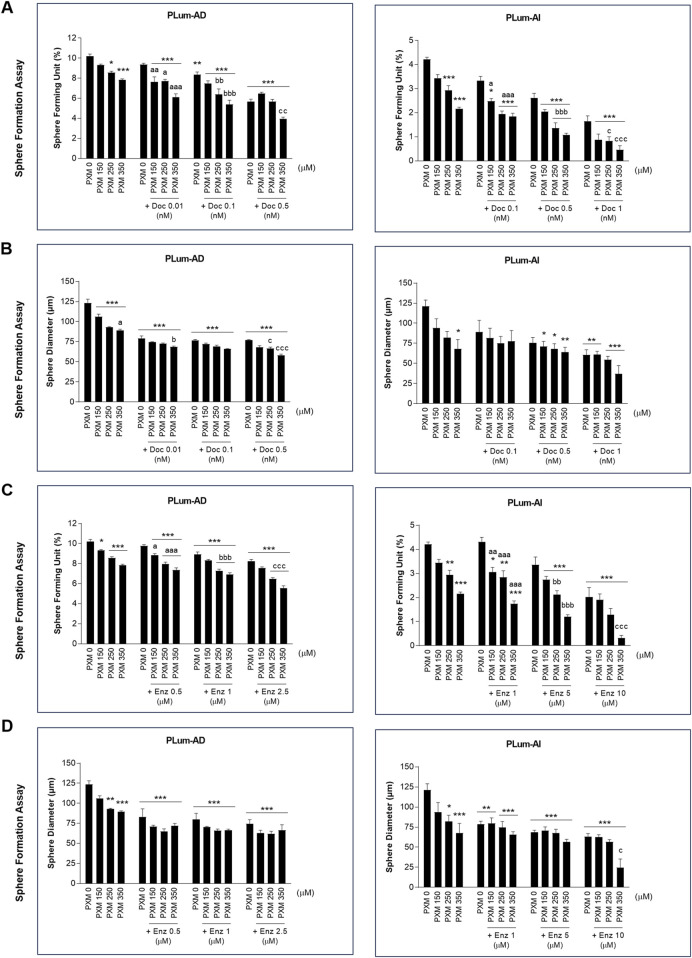
Increasing concentrations of piroxicam, alone or in combination with docetaxel or enzalutamide, reduced the growth of murine prostatospheres in a dose-dependent manner. The effect of 150, 250, and 350 μM of piroxicam on the sphere-forming ability of PLum-AD and PLum-AI cells alone or in combination with the indicated concentrations of docetaxel **(A and B)** or enzalutamide **(C and D)** was assessed using the sphere formation assay. Cells were seeded in Matrigel^TM^ with or without treatment(s) which was replenished every other day. The number of formed spheres was counted 7 to 8 and 4–5 days post-seeding of PLum-AD and PLum-AI cells, respectively. Results are expressed as percentage of sphere-formation unit (SFU) determined based on: SFU= (number of counted spheres/number of input cells) × 100. The average diameter (μm) of at least 30 spheres per condition was determined. Data are reported as mean ± SEM of at least three independent experiments (*P < 0.05; **P < 0.01; ***P < 0.001 indicate significance when comparing to the untreated group, a *P* < 0.05; aa *P* < 0.01; aaa *P* < 0.001 indicate significance when comparing the piroxicam/0.1 nM of docetaxel and piroxicam/1 μM of enzalutamide combination treated groups to 0.1 nM of docetaxel and 1 μM of enzalutamide treated group, respectively, bb *P* < 0.01, bbb *P* < 0.001 indicate significance when comparing the piroxicam/0.5 nM of docetaxel and piroxicam/5 μM of enzalutamide combination treated groups to 0.5 nM of docetaxel and 5 μM of enzalutamide treated group, respectively, c *P* < 0.05; ccc *P* < 0.001 indicate significance when comparing the piroxicam/1 nM of docetaxel and piroxicam/10 μM of enzalutamide combination treated groups to 1 nM of docetaxel and 10 μM of enzalutamide treated group, respectively). PXM: piroxicam; Doc: docetaxel; Enz: enzalutamide.

To assess the effectiveness of PXM, alone or in combination with PCa conventional therapies, in targeting the stem/progenitor cells, *in vitro* 3D sphere formation assay was used. Thus, PLum-AD and PLum-AI cells were cultured in Matrigel^TM^ in the presence of the various treatment conditions for up to 8 and 5 days, respectively. Both untreated murine PCa cells were able to form spheres validating the presence of stem/progenitor sub-population of cells (Figure 7). Interestingly, treatment of PLum-AD and PLum-AI cells with PXM alone inhibited spheres’ growth in a dose-dependent manner (Figures 8A–D). In fact, 350 μM of PXM significantly decreased the percentage of SFU and the average of spheres’ diameter by almost two folds compared to the untreated group in PLum-AI cells (Figures 8A–D).

Importantly, when PXM treatment was combined with Doc or Enz, PLum-AD and PLum-AI spheres’ growth was further significantly reduced compared to the effects of each drug alone (Figures 7, 8). Interestingly, treatment with 350 μM of PXM with either 1 nM of Doc or 10 μM of Enz almost totally inhibited the ability of PLum-AI cells to form spheres (Figures 8A,C).

These results imply that PXM exhibits promising effects in targeting PCa stem/progenitor cells, specifically when combined with PCa standard treatment agents.

### 3.6 PXM reduced the growth of murine cell-derived organoids alone and in combination with Doc or Enz

To investigate the effects of PXM, alone or in combination with Doc or Enz, on the growth of murine organoids, cell-derived organoids was performed. This assay is considered a robust 3D *in vitro* model that more accurately mimics the tumor microenvironment compared to traditional 2D culture or sphere formation assays. By utilizing a higher Matrigel-to-medium ratio and supplementing the culture with growth factors that support the growth of prostate organoids, the assay facilitates the formation of complex 3D structures replicating key features of *in vivo* tumors.

**FIGURE 9:**
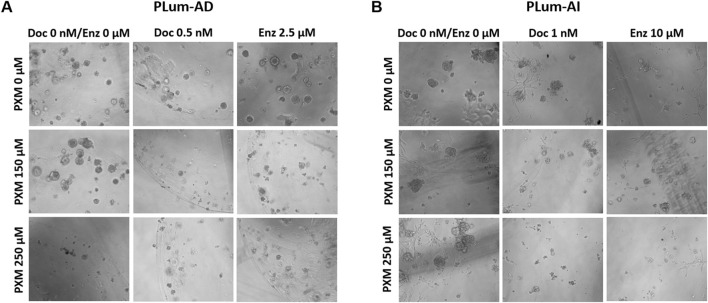
Piroxicam, alone or in combination with docetaxel or enzalutamide, reduced the growth and size of PLum-AD cell-derived organoids. Representative images of PLum-AD **(A)** and PLum-AI **(B)** cell-derived organoids treated with piroxicam and docetaxel or enzalutamide after 7 to 8 and 4–5 days post-seeding, respectively, are shown (scale bar = 100 μm). Images were acquired using Axiovert inverted microscope equipped with Carl Zeiss Zen 2012 image software. PXM: piroxicam; Doc: docetaxel; Enz: enzalutamide.

PLum-AD and PLum-AI cells were suspended in a 9:1 ratio of Matrigel^TM^ to serum-free medium supplemented with essential growth factors and cultured under various treatment conditions. Organoid growth was assessed after 7–8 days for PLum-AD cells and 4–5 days for PLum-AI cells. Untreated cells formed robust 3D organoid structures, confirming their growth potential in this assay (Figure 9).

**FIGURE 10:**
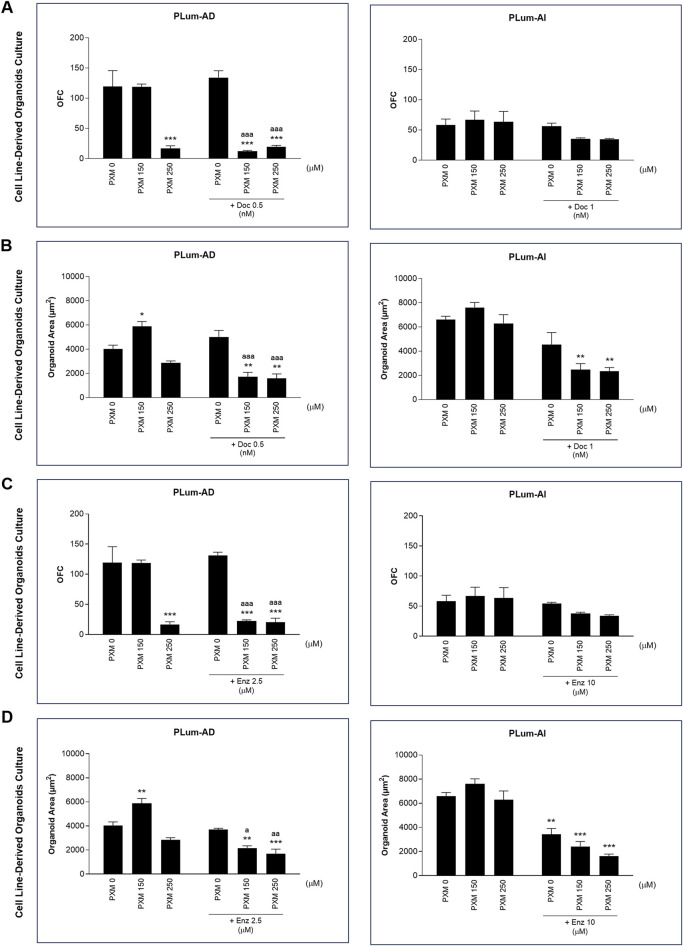
Piroxicam, in combination with docetaxel or enzalutamide, reduced the growth and size of Plum-AD cell-derived organoids. The effect of 150 and 250 μM of piroxicam on the organoid-forming ability of PLum-AD and PLum-AI cells alone or in combination with the indicated concentrations of docetaxel **(A and B)** or enzalutamide **(C and D)** was assessed using the cell-derived organoid formation assay. Cells were seeded in Matrigel^TM^ with or without treatment(s) which was replenished every other day. The number of formed organoids was counted 7 to 8 and 4–5 days post-seeding of PLum-AD and PLum-AI cells, respectively. The OFC was calculated as the number of formed organoids starting with the same number of seeded cells for the different conditions. The average area (μm^2^) of at least 30 organoids per condition was determined. Data are reported as mean ± SEM of at least three independent experiments (*P < 0.05; **P < 0.01; ***P < 0.001 indicate significance when comparing to the untreated group, a *P* < 0.05; aa *P* < 0.01; aaa *P* < 0.001 indicate significance when comparing the piroxicam/0.5 nM of docetaxel and piroxicam/2.5 μM of enzalutamide combination treated groups to 0.5 nM of docetaxel and 2.5 μM of enzalutamide single treated group, respectively). PXM, piroxicam; Doc, docetaxel; Enz, enzalutamide.

Treatment with PXM alone significantly reduced the OFC in PLum-AD cells at 250 μM, while no significant reduction was observed in organoid area. Importantly, a synergistic effect was detected when PXM was combined with either 0.5 nM of Doc or 2.5 μM of Enz, resulting in a marked decrease in both OFC and area in PLum-AD cells (Figures 10A–D). In contrast, the combination of PXM with Doc or Enz in PLum-AI cells led to a slight reduction in OFC without significant effects, and no significant synergistic reduction in organoid area was observed (Figures 10A–D). These findings suggest that PXM, in combination with Doc or Enz, demonstrates a synergistic effect in reducing the organoids’ growth potential in PLum-AD cells but not in PLum-AI cells.

## 4 Discussion

The strategy of repurposing drugs has emerged as a novel approach to explore the anti-cancer potential of existing compounds, aiming to discover new treatment options (Xia et al., 2024b). PCa remains one of the most frequently diagnosed cancers in men and is often associated with poor treatment outcomes. A significant challenge in achieving tumor remission and managing this disease is the development of treatment resistance. In the context of PCa, several drugs have undergone repurposing, with some currently in pre-clinical phases and others in clinical trials (Bahmad et al., 2022). Notably, repurposing NSAIDs has garnered special attention in PCa for their known actions against inflammatory events (Maghsoudi et al., 2023). The latter is well proven to significantly associate with the development and progression of PCa.

In this study, we aimed to investigate the effect of PXM, a nonselective inhibitor of COX enzymes in the NSAIDs category, on sensitizing murine PCa cells to chemotherapy and hormonal therapy drugs. The obtained *in vitro* results provided evidence that PXM enhances PLum-AD and PLum-AI murine PCa cells to Doc and Enz treatments. To the best of our knowledge, this is the first study showing the impact of PXM on these conventional treatment modalities, reflecting the enhanced cytotoxic effect of Doc and Enz on PCa cells in the presence of PXM.

Our findings demonstrate that PXM exhibits significant cytotoxic effects on murine PCa cell lines when administered as a single treatment *in vitro*. In 2D cell culture, treatment with PXM alone reduced the proliferation, viability, and migration of PLum-AD and PLum-AI PCa cells. In 3D Matrigel^TM^-based cell culture, PXM was observed to attenuate the growth of PLum-AD and PLum-AI prostatospheres. Additionally, in the more *in vivo*-relevant cell-line-derived organoid formation assay, PXM suppressed the organoid forming ability and size in PLum-AD cells.

While limited studies have explored the use of PXM in PCa, our findings align with previous research demonstrating the cytotoxic effects of PXM on PCa cells. A study by Kisla et al. showed that PXM increased cell death in PC3 human PCa cells by arresting the cell cycle in the S phase and triggering apoptosis through mitochondrial membrane depolarization and increased caspase levels (SAHINER and BAKAR-ATES, 2022). Another study further proved that PXM had significant cytotoxic potency on PC3 PCa cells, inducing apoptosis by increasing caspase levels and causing mitochondrial membrane potential collapse. The results also demonstrated that optimized PXM core-shell nano-carriers showed significantly improved cytotoxic potency on PC3 cells compared to the pure form of the molecule (Sengel-Turk et al., 2022). Additionally, in an *in vivo* PCa animal model, treating rats with PXM showed to suppress to some extent tumor growth, bone destruction, and metastasis without evidence of toxicity (Pollard and Luckert, 1986). In our study, we also tested PXM in three human prostate cancer cell lines as part of our preliminary experiments (Supplementary Figure 1). Our results showed that PXM exhibited a cytotoxic effect on the proliferation of LNCaP, DU145, and PC3 cells in a dose-dependent manner.

Importantly, when combining PXM with PCa conventional treatments, Doc or Enz, synergistic effects were clearly observed. These augmented anti-cancer effects validate findings from other studies highlighting the potential of NSAIDs, particularly PXM, to enhance the efficacy of conventional cancer therapies and overcome therapy resistance and toxicity. However, no studies have evaluated the combinatory effects of PXM with conventional treatments in PCa. In a study involving bladder cancer cell lines, a combinatorial drug exposure of PXM and Carboplatin produced a significant synergistic reduction in cell growth compared to single-drug treatment. This combination also led to notable increases in morphological alterations, a marked decrease in the Ki-67 proliferative marker, a considerable enhancement of autophagic vacuoles, and minimal effects on apoptosis in both cell lines (Silva et al., 2017b). Moreover, testing PXM and cisplatin in a mouse model of peritoneal mesothelioma revealed marked tumor growth inhibition and extended survival of mice treated with a combination of these two drugs. This suggested that PXM sensitizes mesothelioma cells to cisplatin-induced cytotoxicity by modulating the expression of specific target genes (Spugnini et al., 2006).

Interestingly, our *in silico* analysis of murine PCa models revealed an enrichment of inflammation-related pathways during the progression of PCa from androgen-dependent to androgen-independent aggressive stages. This underscores the suitability of our cell models, PLum-AD and PLum-AI, for studying NSAIDs. Additionally, protein-protein interaction network analysis of DEGs) identified interactions among signaling molecules within inflammatory pathways, with IL6 emerging as a central player in all interactions. The latter is identified as an oncogenic factor associated with PCa growth and progression (Culig and Puhr, 2018). These findings validate the use of our cell models as a robust platform for exploring the potential anti-cancer effects of NSAIDs in PCa.

We acknowledge several limitations in our study. Firstly, we utilized a restricted number of PCa cell lines *in vitro*, and incorporating additional cell lines with diverse genetic backgrounds and aggressiveness could enhance the comprehensiveness of our findings. Additionally, inclusion of a normal cell line would provide further evidence regarding the safety of our combinatory treatment approach. Further studies are warranted to evaluate the molecular impact of our treatment and elucidate its underlying mechanisms and downstream signaling effectors, particularly concerning inflammatory pathways, in both 2D and 3D cell culture systems. To better characterize the effects of PXM treatment at the transcriptional level on PCa cells *in vitro*, transcriptomic analyses can be done to determine the treatment-modulated genes. Finally, additional *in vivo* or *ex vivo* investigations are essential to replicate our findings in a more clinically relevant cancer model.

In conclusion, our study adds to the growing literature on the anti-cancer properties of NSAIDs. Our work provides evidence supporting the repurposing of PXM as a potential adjunctive therapy for PCa. Through sensitizing PCa cells to conventional treatments, PXM holds promise in improving treatment outcomes and overcoming drug resistance. Further research, including preclinical and clinical studies, is warranted to validate the efficacy and safety of PXM-based combination therapies in PCa patients. Ultimately, the repurpose of PXM demonstrates the importance of exploring alternative treatment strategies to tackle cancer and improve therapeutic outcomes in PCa patients.

## Data Availability

The datasets presented in this study can be found in online repositories. The names of the repository/repositories and accession number(s) can be found in the article/supplementary material.
