# Supplementary figures and images for: Repurposing piroxicam enhances the antineoplastic effects of docetaxel and enzalutamide in prostate cancer cells using 2D and 3D in vitro culture models
Source: Front Cell Dev Biol. 2025 Jul 1;13:1551010. doi: 10.3389/fcell.2025.1551010 (PMC12259556; doi:10.3389/fcell.2025.1551010)

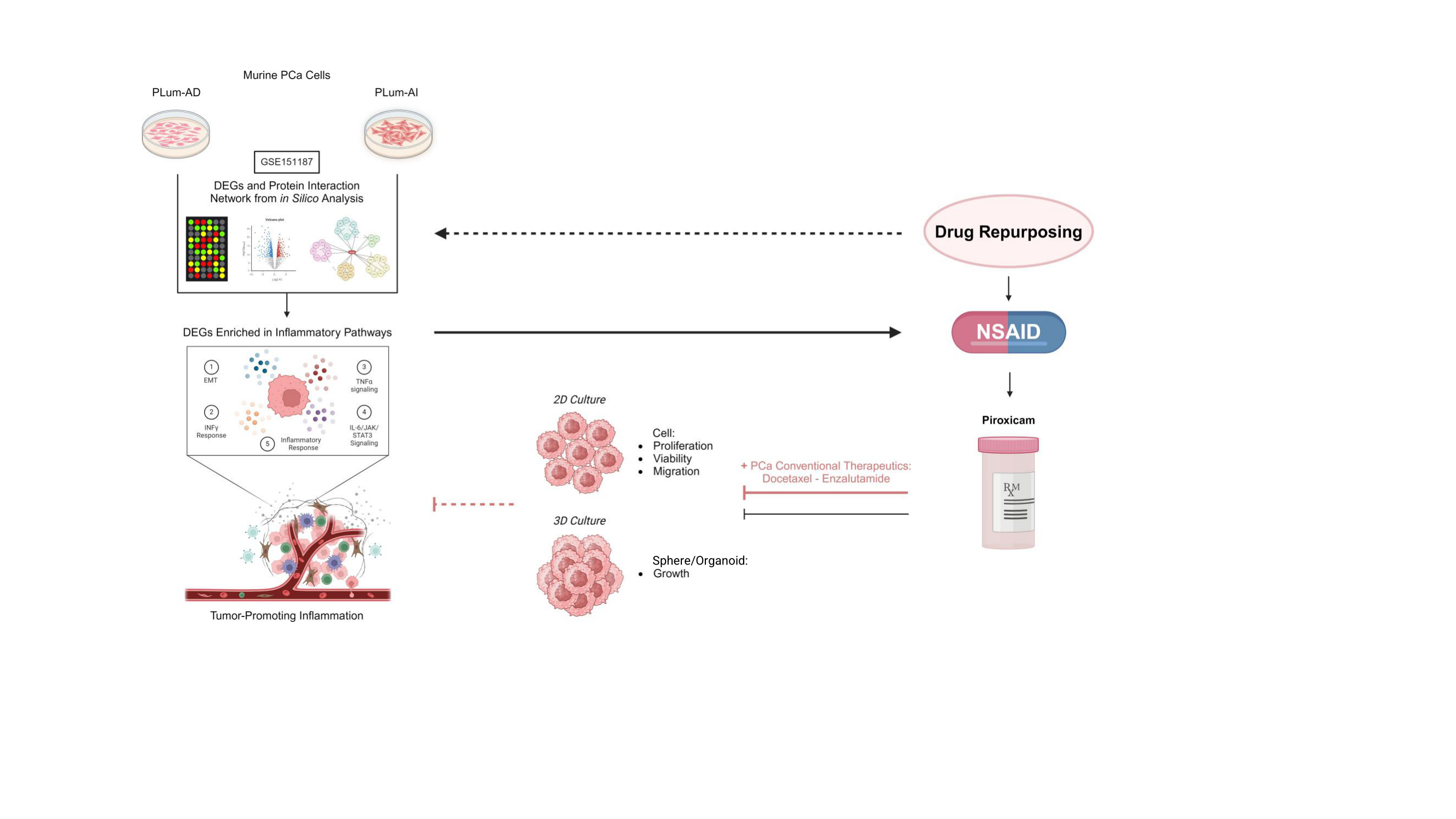

Supplement: Supplementary file 2 [file Image1.tiff]
